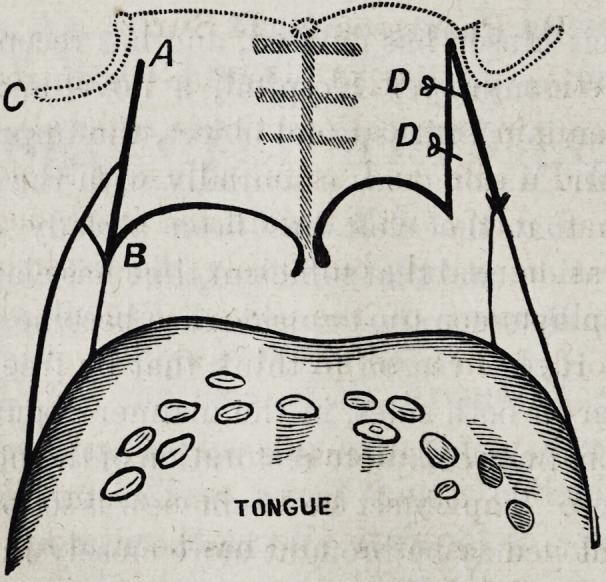# Case of Cleft Palate

**Published:** 1870-07

**Authors:** 


### SELECTED ARTICLES.

### ARTICLE VI.

### *Case of Cleft Palate.*

**NOVEL PROCEDURE FOR IMPROVING THE VOICE AFTER THE OPERATION OF STAPHYLORAPHY.**

(Under the care of Mr. Francis Mason.)

Every surgeon who has any experience of congenital cleft palate will, we believe, admit that the principal object for which the operation is usually undertaken—that is, the improvement of the voice,—is seldom attained. In the most favorable cases, however accurately the edges of the cleft may be adjusted, and however well satisfied the operator may be with the result of his manipulative skill, the patients and their friends invariably express some, and often a great deal of disappointment that the tone of the voice remains much as it was. A little consideration will suffice to show why the expected improvement does not take place. It should be remembered, that the condition of congenital cleft palate is not that of a mere slit or rent, but is an absolute want of substance in the part; and, in order to make good this deficiency, a "gusset," as it is termed, is required. Now to make a gusset of living material seems difficult, if not impracticeable; hence surgeons are contented simply to bring the edges of the cleft together, union being almost infallible if the muscles be divided in accordance with the plan recommended by Sir Wm. Fergusson. The result of the operation is that the two sides are neccessarily put considerably on the stretch, and the soft palate, instead of being freely movable and capable of being brought by muscular action upwards and backwards against the posterior wall of the pharynx, remains as a tight curtain, so placed as to allow air to pass into the posterior nares, causing that peculiar nasal twang more or less constantly noticed in this condition. It therefore stands to reason the voice cannot be materially altered or improved under such circumstances.

Mr. Francis Mason has devised, and has recently carried out at the Westminister Hospital, a novel and ingenious proceeding, having for its special object, the improvement of the voice. Mr. Mason deals essentially with the soft palate, and assumes that the cleft has been already closed by a previous operation, and that sufficient time has elapsed so that the circulation between the two sides has become thoroughly established. He has reason to think that by freely dividing the soft palate on both sides, in the manner about to be described, partial, if not complete restoration of the normal voice may be effected. The object he has in view is so to release the soft palate that it may be brought more closely in apposition with the back part of the pharynx, and thus to a certain extent if not wholly, do away with the disagreeable nasal twang already alluded to. The subjoined diagrammatic wood-cut, representing the isthmus of the fauces, seen from the front will serve to illustrate the steps of this operation. The dotted lines indicate the edge of the hard palate and the hamular process on each side. The cicatrices of the suture. in the middle line of the soft palate, the result of the previous operation, are also shown.

The patient is placed in the recumbent posture, and may or may not take chloroform. The soft palate is completely transfixed with a sharp pointed knife at A, at the inner edge of the hamular process (C), the outline of which may, in most cases, be distinctly seen in the living subject. The incision is carried downwards from A to B, dividing the whole of the soft palate. A similar incision is made on the opposite side, and the operation is completed by introducing with a needle in a handle, one or more sutures from before backwards at D, hemming (so to speak) the anterior and posterior mucous edges, thus preventing the newly-made raw surfaces from uniting by granulation. In this proceeding the palato-glossus, palato-pharyngeus, and the tensor palati muscles are purposely divided. The levator palati is not interfered with, or at most only a few of its fibres are cut.

The case upon which Mr. Mason operated is that of the boy, now aged six, whose palate he closed without chloroform in October last (see the *Lancet*, Jan. 9th, 1869). The result is so far satisfactory that there is a decided improvement in the boy's voice. As this is the first patient upon whom this operation has been performed, it would be premature to form a strong opinion as to the probable results; meanwhile the operation seems to commend itself as being extremely simple, free from danger to important vessels, and not particularly painful—a lump of ice in the mouth for a minute or so would render it almost painless. Moreover, chloroform may be administered (as in this case), vomiting, if it occur, being no disadvantage. There is an ample supply of blood to this part so that sloughing need not be apprehended; and further it is, a proceeding that may be undertaken at any period of life, and repeated as often as necessary, in case the cut surfaces should unite by granulation. Mr. Mason contends that it can do no harm; and if, on the other hand, it be attended with even partial success, it supercedes any mechanical appliance, and confers an inestimable boon on patients whose condition is distressing both to themselves and to those with whom they associate.—*Lancet.*

---

## Figures and Tables

**Figure f1:**